# Wearable powered overground exoskeleton reduces dose-dependently the spasticity in individuals with spinal cord injury

**DOI:** 10.1038/s41393-026-01181-6

**Published:** 2026-03-09

**Authors:** Sanaz Pournajaf, Giovanni Morone, Giorgio Felzani, Leonardo Pellicciari, Elena Sofia Cocco, Carlotta Maria Manzia, Laura Simoncini, Rocco Salvatore Calabrò, Marco Franceschini

**Affiliations:** 1 Research Area in Neuromotor Rehabilitation and Rehabilitation Robotics, Department of Neuroscience, IRCCS San Raffaele Roma, Rome, Italy; 2 Department of Mental and Physical Health and Preventive Medicine, University of Campania ‘Luigi Vanvitelli’, Naples, Italy; 3 Department of Life, Health and Environmental Sciences, University of L’Aquila, 67100 L’Aquila, Italy; 4 San Raffaele Institute of Sulmona, 67039 Sulmona, Italy; 5 IRCCS Istituto delle Scienze Neurologiche di Bologna, Bologna, Italy; 6 Spinal Unit, Montecatone Rehabilitation Institute, Imola, Italy; 7 IRCCS Centro Neurolesi Bonino-Pulejo, 98124 Messina, Italy

**Keywords:** Spinal cord diseases, Outcomes research

## Abstract

**Study design:**

A multicenter retrospective pre-post cohort study.

**Objective:**

This study aimed to evaluate the dose-dependent effects of overground exoskeleton-assisted gait training on spasticity and functional independence in individuals with SCI.

**Setting:**

IRCCS San Raffaele Rome, San Raffaele Sulmona, Centro Neurolesi Bonino Pulejo IRCCS in Messina, and Montecatone Rehabilitation Institute in Imola.

**Methods:**

Seventy-four participants with SCI underwent a minimum of five gait training sessions using the Ekso™ exoskeleton. Primary outcomes included changes in spasticity, measured via the aggregated Modified Ashworth Scale (MAS), and functional independence, assessed by the Spinal Cord Independence Measure (SCIM) III. Secondary analyses explored the influence of treatment dosage.

**Results:**

Significant reductions in spasticity were observed across all muscle groups (proximal, intermediate, and distal) post-intervention (p < 0.0001). SCIM scores also showed substantial improvements (p < 0.0001), indicating enhanced functional independence. Participants receiving ≥15 sessions demonstrated greater gains compared to those with fewer sessions (p < 0.05).

**Conclusions:**

Overground exoskeleton-assisted gait training effectively reduces spasticity and improves functional independence in individuals with SCI, with greater benefits observed at higher session frequencies. These findings support the integration of wearable powered exoskeletons into standard rehabilitation protocols for SCI.

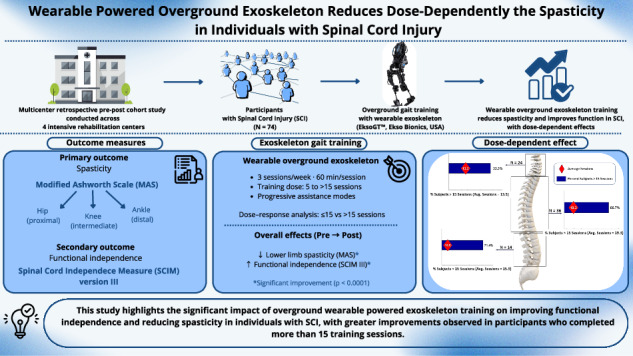

## Introduction

Spinal Cord Injury (SCI) is a severe condition leading to significant motor and sensory deficits, particularly affecting mobility and ambulation. Among its secondary complications, spasticity is one of the most prevalent and disabling, requiring targeted rehabilitation strategies [1, 2]. Recent advancements in robotic-assisted rehabilitation, particularly with wearable powered overground exoskeletons, offer a novel approach to gait training in individuals with SCI [3, 4]. These devices not only assist in restoring walking ability but have also shown potential in modulating muscle tone and reducing spasticity [5–7]. Recent studies have highlighted the feasibility and effectiveness of using exoskeletons in inpatient rehabilitation settings, demonstrating the utilization of overground exoskeleton gait training during inpatient rehabilitation and its positive outcomes in improving mobility and independence [8]. Furthermore, Baunsgaard et al. conducted a large cohort study that evaluated the safety and gait outcomes of gait training after SCI, further supporting the integration of these devices into clinical rehabilitation programs [9].

Unlike stationary robotic-assisted devices, which are classified as device-in-charge robots [10], overground exoskeletons function as patient-in-charge systems, allowing individuals to walk in real-world environments and experience a more dynamic and physiologically typical gait [11]. This type of approach requires more active participation from the patient during walking, including a higher cognitive demand, which may further enhance neuroplasticity and motor learning [12]. These exoskeletons facilitate repetitive, high-intensity gait training, essential for functional recovery post-SCI [13]. The repetitive nature of movement promotes cortical reorganization, a key mechanism underlying neuroplasticity and functional improvement in individuals with spinal cord injury [14].

While the concept of anatomical spinal cord repair remains speculative and is not directly evidenced by current clinical neurophysiological data, functional gains following exoskeleton-assisted gait training are believed to arise from adaptive plasticity within the sensorimotor system. Functional MRI studies have demonstrated that, after spinal cord injury, sensorimotor activation patterns initially expand and later become more focused in cortical motor areas as recovery progresses [15]. Moreover, paired-pulse somatosensory evoked potential (ppSEP) protocols have shown normalization of cortical excitability in patients after exoskeleton-assisted gait training, supporting the hypothesis of cortical-level adaptation [16]. These neuroplastic changes may contribute to improved voluntary motor control and walking ability. Additionally, exoskeleton-assisted walking has been associated with psychological benefits, including increased motivation and improved mood, which may further enhance engagement in the rehabilitation process [17, 18]. Overground exoskeletons provide dynamic support, adapt to user needs in real-time, and deliver proprioceptive stimuli that simulate natural gait, promoting sensory feedback essential for neuromuscular re-education [18, 19]. Their design and feedback mechanisms not only facilitate muscle engagement but also improve postural demand and trunk activation by requiring the user to perform dynamic weight shifts to maintain continuous walking.This interaction with the exoskeleton device enhances the rehabilitation experience, supporting balance recovery and functional gait patterns [20]. By replicating near-physiological gait, these exoskeletons help optimize motor learning and neuroplasticity, which are critical for restoring mobility after SCI [13]. Even in chronic SCI, the spinal cord retains a latent capacity for plasticity, particularly within spinal interneuronal networks, reflex pathways, and central pattern generators (CPGs). Robotic gait training can reactivate these mechanisms through repeated, task-specific sensory and motor stimulation[21, 22].

However, there is growing evidence that such devices can also improve non-motor functions, including spasticity and quality of life. Reductions in spasticity have been associated with enhanced comfort and participation in daily activities, while improvements in psychological well-being and perceived quality of life reflect broader rehabilitation benefits beyond gait parameters. In light of this, we deliberately chose to focus also on these non-motor domains and selected outcome measures that better capture these dimensions of recovery. Recent studies have shown such effects in both spinal cord injury and stroke populations. For example, Juszczak et al. [23] demonstrated significant reductions in spasticity after exoskeleton-assisted training in individuals with SCI, while van Nes et al. [24] reported significant improvements in several quality-of-life domains, including pain, mental health, and social functioning, following an 8-week exoskeleton intervention. At the same time, De Luca et al. also showed an improvement of constipation and quality of life in people with stroke undergoing RAGT with the Ekso-GT [25].

Despite these promising advancements, limited research has specifically investigated the dose-dependent effects of overground exoskeleton training on spasticity reduction in individuals with SCI. While some studies have explored the impact of robotic gait training on mobility and spasticity, the role of treatment dosage and its correlation with functional improvements remains insufficiently understood [9, 26]. Therefore, the primary objective of this study was to evaluate the effect of gait training using a wearable-powered exoskeleton on spasticity and functional independence in individuals with SCI. The secondary purpose was to explore treatment dose in terms of the number of sessions as a potential influencer of rehabilitation outcomes to provide robust clinical evidence to guide best practices for integrating robotic therapy into standard care for individuals with SCI, addressing a critical unmet need in rehabilitation. This study contributes to the growing body of evidence supporting the integration of exoskeletons into SCI rehabilitation, addressing a critical gap in knowledge regarding the interplay between robotic gait training, spasticity management, and functional independence.

## Methods

This multicenter retrospective pre-post cohort study was conducted across four intensive rehabilitation centers (IRCCS San Raffaele Rome, San Raffaele Sulmona, Centro Neurolesi Bonino Pulejo IRCCS in Messina, and Montecatone Rehabilitation Institute in Imola) between October 2021 and October 2023.

This study protocol, adhering to the Declaration of Helsinki principles, was approved by the Local Ethics Committee (CET Lazio Area 5) on July 03, 2024, with the code number RP- 120/SR/24. Upon admission, all participants provided written informed consent for the collection and use of their data in the database.

Eligible participants were adults aged 18–70 years with a confirmed diagnosis of SCI and the ability to maintain an upright posture for at least one minute with sufficient cardiovascular stability, including those able to stand with double or single support, with or without supervision, or with a minimum-to-moderate level of therapist assistance, based on clinical judgment (i.e., not fully dependent on manual support). Participants included both inpatients undergoing intensive rehabilitation and outpatients attending scheduled gait training sessions. All participants were clinically stable and maintained their pharmacological therapy unchanged throughout the observation period. Inclusion criteria required a minimum of five training sessions using a wearable-powered exoskeleton [27], with a frequency of two or more sessions per week. Exclusion criteria included neurological comorbidities, contraindications to exoskeleton use, botulinum toxin in the previous 3 months and during the observation phase, cognitive deficits impairing comprehension or participation, or severe comorbidities with relevance to cardiovascular function or physical limitations that prevented exoskeleton fitting [28].

Participants underwent gait rehabilitation training with the EksoGT™ wearable overground powered exoskeleton (Ekso Bionics, Richmond, CA, USA) as part of their daily standard multidisciplinary rehabilitation program, tailored to the clinical and functional status of each participant. This program also included conventional physiotherapy, occupational therapy, and other individualized rehabilitation or physical activity interventions according to each center’s multidisciplinary plan. Participants included both inpatients undergoing intensive rehabilitation and outpatients attending scheduled gait training sessions. All participants were clinically stable and maintained their pharmacological therapy unchanged throughout the observation period. Gait training sessions with the exoskeleton were conducted three times per week, each lasting 60 min, and were integrated into the personalized rehabilitation plan. At the beginning of each session, the exoskeleton was fitted and calibrated to ensure optimal alignment and weight distribution. The EksoGT™ software system was used to adjust stride length, step height, and cadence based on the user’s anthropometrics and motor capabilities. Participants initially trained in Bilateral Max Assist mode, where the exoskeleton provided full robotic assistance for hip and knee flexion-extension throughout the gait cycle. As training progressed, the level of assistance was gradually reduced, transitioning to Adaptive mode, in which the exoskeleton dynamically modulated support in response to the user’s voluntary movement. Further progression involved ProStep+ mode, which required participants to actively initiate each step, with the exoskeleton providing adaptive assistance in response to the user’s effort. This structured, progressive training approach was designed to facilitate gradual engagement in voluntary stepping, promoting motor learning and neuromuscular adaptation. Throughout the intervention, the EksoGT™ system continuously monitored gait parameters, enabling therapists to fine-tune training intensity based on each participant’s spasticity response, motor control, and overall functional progression. None of the patients used the exoskeleton outside of prescribed treatments.

The primary outcome measure was changes in overall lower limb spasticity, measured with the aggregated modified Ashworth Scale (MAS).

Scores of 1, 1 + , 2, 3, and 4 (translated into consecutive values from 1 to 5) reflect progressively increasing levels of observed spasticity [29].

For descriptive purposes, the “more affected side” was defined as the limb presenting the higher MAS score at baseline, whereas the “less affected side” was defined as the contralateral limb with a lower baseline MAS score. These definitions were applied consistently across joints.

In detail, similarly to study of [9] the spasticity of the hip, knee and ankle flexor and extensors referred as proximal, intermedium and distal, respectively has been evaluated with MAS. The sum-score values were in the lower end of the 0–30 sum-score scale for each leg (less affected, and more affected). The reason for this was the presence of some subjects with MAS ratings having a value of 0, i.e. no spasticity, since all values of the 6 muscle groups per leg, were included in the sum-score (i.e. mitigating floor effect); and for a clinical aim: to match site of the lesion with potential different tone modification in different muscle groups. Changes in functional independence, measured using the Spinal Cord Independece Measure (SCIM) version III, were considered as the secondary outcome [30]. The SCIM assesses traumatic and non-traumatic, acute and chronic activities of daily living (coordination, eating, functional mobility, incontinence) of persons with a spinal cord injury. The SCIM III is composed of 19 items that assess 3 domains (Self-care; Respiration and Sphincter Management; Mobility), and the total scores range from 0 (lower participation in ADL) to 100 (higher participation in ADL). Due to the retrospective nature of the dataset, only the total SCIM III score was consistently available across all centers; therefore, subscale-level analyses could not be performed.

Demographic and clinical data were collected for all participants, including age (in years), gender (male or female), and the etiology of spinal cord injury (traumatic or non-traumatic). Baseline neurological status was assessed using the American Spinal Injury Association (ASIA) Impairment Scale, ranging from grade A (complete injury) to grade E (normal). Lesion level was categorized as cervical (C3–C7), thoracic (D1–D12), or lumbar (L1–L3). Time since the acute event was recorded and classified as either early phase (<6 months) or late phase (≥6 months) for analytical purposes and to avoid inconsistency with established SCI terminology [31]. Clinical outcome measures, including the Modified Ashworth Scale (MAS) and the Spinal Cord Independence Measure (SCIM) III, were recorded at baseline (T0) and at the end of treatment (T1), as part of standard clinical practice. The treatment dosage was also documented, defined as the total number of exoskeleton-assisted gait training sessions completed. Additionally, any adverse events related to the use of the exoskeleton—whether clinically evaluated or patient-reported—were systematically recorded. All data were anonymized and transferred to the lead center (IRCCS San Raffaele, Rome) for analysis.

Information on motor Zone of Partial Preservation (ZPP) was not consistently available across centers due to the retrospective nature of the dataset and was therefore not included in the analyses.

Descriptive statistics were calculated to describe the collected variables; particularly, including mean ± standard deviation (SD), median with first and third quartile, and frequency with percentage were computed for intervallic, ordinal and categorical variables, respectively. A paired t-test was used to evaluate pre- and post-training changes in SCIM and MAS scores. To study the difference on pre- and post-training changes between sub-groups according to the median session of treatments, time since the acute event (i.e., early phase [less than 6 months] and late phase [over 6 months]), etiology (traumatic, non-traumatic), lesional level (i.e., cervical, dorsal, lumbar), and etiology (traumatic, non-traumatic), secondary analysis with paired t-test was performed. Moreover, the mean change with its 95% confidence interval was computed for each comparison. Any differences regarding the clinical variables (i.e., etiology, ASIA at baseline, lesional level, and time since the acute event) at the baseline between the subgroups were assessed by the means of a chi-squared test. All statistical analyses were run with SPSS software (version 21 for Windows, SPSS Inc., Chicago, IL; 2004). Alpha value was set for p < 0.05.

## Results

A total of 74 individuals with SCI (mean ± SD age: 48.3 ± 15.3 years; 78.4% male; 67.6% traumatic etiology) was included in this study. Table 1 shows the demographic and clinical characteristics of the sample at baseline.

**Table 1:** Main demographic and clinical characteristic of the included individuals (N = 74).

Variable	Value
**Age,** *years*	48.3 ± 15.3
**Gender**	
Male	58 (78.4%)
Female	16 (21.6%)
**Etiology**	
Traumatic	50 (67.6%)
Non-traumatic	24 (32.4%)
Degenerative conditions	6 (8.1%)
Vascular disorders	5 (6.8%)
Tumors	4 (5.4%)
Spinal arteriovenous fistula	4 (5.4%)
Post Surgery	3 (4.0%)
Infections	2 (2.4%)
**ASIA at baseline**	
A	15 (20.3%)
B	19 (25.7%)
C	24 (32.4%)
D	16 (21.6%)
E	0 (0.0)
**Lesional level**	
Cervical (C3-C7)	24 (32.4%)
Dorsal (D1-D12)	36 (48.6%)
Lumbar(L1-L3)	14 (18.9%)
**Time since acute event**	
Early phase (<6 months)	34 (45.9%)
Late Phase (≥6 months)	40 (54.1%)
**Number of sessions with exoskeleton**	16.8 ± 0.8.8

*NLI* neurological level injury, *LOS* length of stay.

Values are reported ad frequency (percentage) or mean ± standard deviation.

Detailed results for the study outcomes are reported in Table 2.

**Table 2:** Differences between the baseline assessment and the discharge (N = 74).

Outcome measures	T0	T1	t-score*	p-value*	Mean change [95% CI]
SCIM III	40.8 ± 22.9	57.2 ± 24.5	−4.606	**<0.0001**	−16.4 [−23.3, −9.4]
Proximal MAS	4.1 ± 4.7	3.1 ± 3.3	3.522	**0.001**	1.1 [0.5, 1.6]
Intermedium MAS	4.0 ± 4.3	3.0 ± 3.2	3.754	**<0.0001**	1.0 [0.5, 1.5]
Distal MAS	5.0 ± 4.7	3.4 ± 3.4	5.56	**<0.0001**	1.6 [1.1, 2.2]
Aggregated MAS	13.2 ± 12.9	9.5 ± 8.8	5.006	**<0.0001**	3.7 [2.2, 5.1]

*CI* confidence interval, *SCIM III* Spinal Cord Independence Measure (Version III), *MAS* modified Ashworth Scale.

Value is presented as mean ± standard deviation. Significant p-values are reported in bold. * Related to the paired t-test.

Data analysis revealed significant improvements in aggregated MAS scores between the pre- and post-treatment assessments which demonstrated a significant reduction in proximal (p = 0.001; mean change = 1.1 points), intermedium (p < 0.0001; mean change = 1.0 points), distal (p < 0.0001; mean change = 1.6 points) and as a result a total score (p < 0.0001; mean change = 3.7 points) suggesting a marked reduction in spasticity post-intervention. In particular, 16 participants having no spasticity at baseline didn’t gain any change after treatment. While, of the rest of the sample (N = 58) 70.7% (N = 41) has gained a spasticity reduction by ≥1). Also, SCIM scores have demonstrated an improvement (p < 0.0001; mean change = −16.4 points), indicating notable gains in functional independence across the participants (Table 2).

**Fig. 1:**
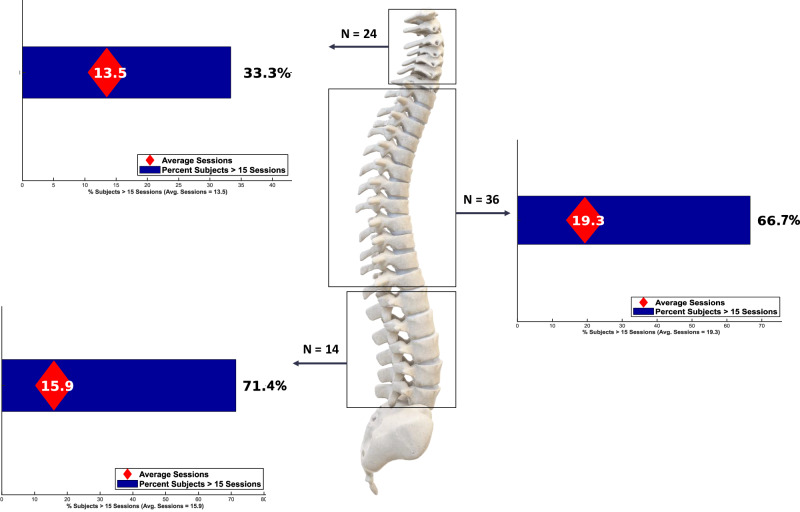
Distribution of Individuals with SCI Completing Over 15 Gait Training Sessions, Categorized by Lesion Level.

The median number of treatment sessions for the included subjects was 15. Participants with fewer than 15 sessions showed less improvement compared to those who completed more than 15 sessions (Table 3). No difference were found between fewer than 15 sessions and more than 15 sessions subgroups considering the etiology (i.e., traumatic and non-traumatic; χ^2^ = 0.123, p = 0.726), ASIA at baseline (i.e., A, B, C, D; χ^2^ = 5.355, p = 0.148), time since the acute event (i.e., early phase, late phase; χ^2^ = 3.244, p = 0.072), and lesional level (i.e., cervical, dorsal, lumbar; χ^2^ = 5.437, p = 0.066). Figure 1 illustrates the percentage of individuals with SCI who completed more than 15 gait training sessions using an overground powered exoskeleton, categorized by lesion levels (cervical, thoracic, and lumbar). The data suggest that lower lesion levels are associated with a higher percentage of individuals completing a greater number of gait training sessions.

**Table 3:** Secondary analysis considering the session treatment, investigating differences between the baseline assessment and the discharge.

Sessions	Outcome measures	T0	T1	t-score*	p-value*	Mean change [95% CI]
Less or equal to 15 sessions (N = 41)	SCIM III	44.5 ± 23.5	53.3 ± 27.5	−1.874	0.068	−8.8 [−18.0, 0.4]
	Proximal MAS	4.0 ± 4.6	3.2 ± 3.3	2.022	0.050	0.8 [0.0, 1.6]
	Intermedium MAS	3.7 ± 4.3	2.9 ± 3.3	2.044	**0.048**	0.8 [0.1, 1.5]
	Distal MAS	4.7 ± 5.0	3.3 ± 3.6	3.488	**0.001**	1.4 [0.6, 2.1]
	Aggregated MAS	12.4 ± 13.0	9.4 ± 8.8	2.876	**0.006**	3.0 [0.9, 5.0]
Over 15 sessions (N = 33)	SCIM	36.2 ± 21.7	61.9 ± 19.6	−5.120	**<0.001**	−25.7 [−35.6, −15.9]
	Proximal MAS	4.3 ± 5.0	2.9 ± 3.2	3.039	**0.005**	1.3 [0.5, 2.2]
	Intermedium MAS	4.5 ± 4.2	3.2 ± 3.2	3.540	**0.001**	1.2 [0.6, 1.9]
	Distal MAS	5.4 ± 4.5	3.4 ± 3.3	4.422	**<0.001**	2.0 [1.1, 2.9]
	Aggregated MAS	14.2 ± 12.9	9.6 ± 8.9	4.419	**<0.001**	4.6 [2.6, 6.6]

*CI* confidence interval, *SCIM III* spinal cord independence measure (Version III), *MAS* modified ashworth scale.

Value is presented as mean ± standard deviation. Significant p-values are reported in bold. * Related to the paired t-test.

**Fig. 2:**
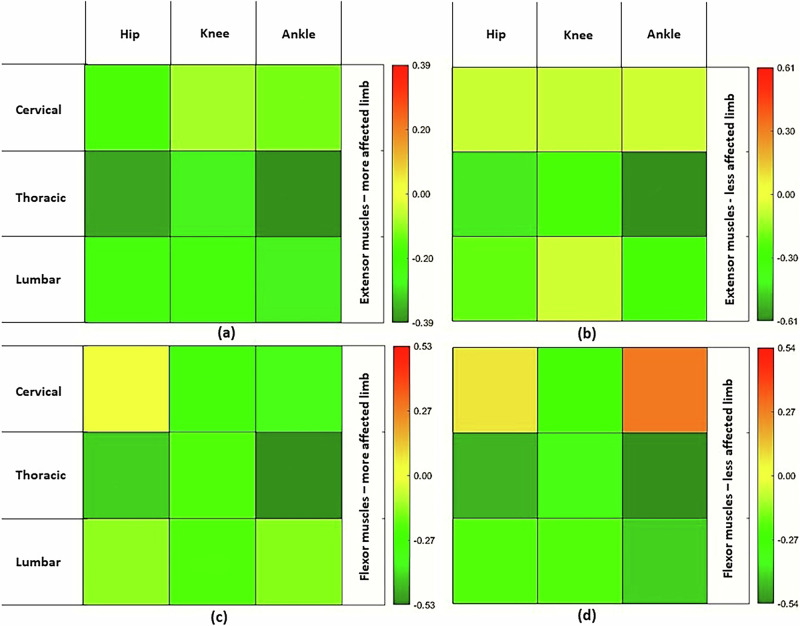
Gradient-based heatmap of spasticity variations across the analyzed muscle groups. Each grid displays a gradient from green to red, representing the delta variation of the Modified Ashworth Scale (MAS) categorized by lesion site and anatomical muscle groups. Darker green shades indicate greater improvements in MAS scores, while redder shades signify less improvement. The figure also differentiates between the more affected and less affected side, as well as between flexor and extensor muscles (**a**), (**b**), (**c**), (**d**).

To visualize the distribution of spasticity changes across different muscle groups, a gradient-based heatmap was generated (Fig. 2). The colors represent the magnitude of MAS variation, with darker green indicating greater spasticity reduction and red representing minimal or no change. The grid also distinguishes between the more and less affected limbs, as well as between flexor and extensor muscles.

The subgroup analysis for time since the acute event suggested that participants in the early phase reported less improvement in terms of function and spasticity compared to ones in the late phase (Table 4). No difference was found between early and late phase subgroups considering the etiology (i.e., traumatic and non-traumatic; χ^2^ = 0.235, p = 0.628), ASIA at baseline (i.e., A, B, C, D; χ^2^ = 2.315, p = 0.510), and lesional level (i.e., cervical, dorsal, lumbar; χ^2^ = 0.972, p = 0.615).

**Table 4:** Secondary analysis considering the time since the acute event (i.e., early phase [less than 6 months] and late phase [over 6 months]), investigating differences between the baseline assessment and the discharge.

Times since the acute event	Outcome measures	T0	T1	t-score*	p-value*	Mean change [95% CI]
Eraly Phase (N = 34)	SCIM III	30.5 ± 20.3	55.1 ± 26.3	−3.899	**<0.001**	−24.7 [−37.1, −12.3]
	Proximal MAS	2.3 ± 4.3	2.1 ± 2.9	0.632	0.532	0.3 [−0.6, 1.1]
	Intermedium MAS	2.6 ± 4.1	1.9 ± 2.9	1.518	0.138	0.6 [−0.2, 1.5]
	Distal MAS	3.6 ± 4.4	2.3 ± 2.9	3.374	0.002	1.3 [0.5, 2.0]
	Aggregated MAS	8.5 ± 12.1	6.3 ± 7.8	2.075	0.046	2.3 [0.2, 4.4]
Late Phase (N = 40)	SCIM III	49.6 ± 21.5	58.9 ± 23.1	−2.673	0.011	−7.5 [−14.9, −0.20]
	Proximal MAS	5.7 ± 4.6	4.0 ± 3.3	4.327	**<0.001**	1.7 [0.9, 2.5]
	Intermedium MAS	5.3 ± 4	4.0 ± 3.2	3.957	**<0.001**	1.2 [0.5, 1.8]
	Distal MAS	6.3 ± 4.7	4.3 ± 3.7	4.433	**<0.001**	1.7 [0.9, 2.6]
	Aggregated MAS	17.2 ± 12.3	12.3 ± 8.7	5.002	**<0.001**	4.6 [2.6, 6.6]

*CI* confidence interval, *SCIM III* spinal cord independence measure (version III), *MAS* modified Ashworth Scale.

Value is presented as mean ± standard deviation. Significant p-values are reported in bold. * Related to the paired t-test.

The subgroup analysis for lesional level suggested that dorsal participants reported more improvement in terms of function and spasticity compared to cervical and lumbar participants (Table 5). No difference was found between cervical, dorsal and lumbar subgroups considering the etiology (i.e., traumatic and non-traumatic; χ^2^ = 1.005, p = 0.605), ASIA at baseline (i.e., A, B, C, D; χ^2^ = 10.668, p = 0.099), and time since the acute event (i.e., early phase, late phase; χ^2^ = 0.972, p = 0.615).

**Table 5:** Secondary analysis considering the lesional level (i.e., cervical, dorsal and lumbar), investigating differences between the baseline assessment and the discharge.

Etiology	Outcome measures	T0	T1	t-score*	p-value*	Mean change [95% CI]
Cervical (N = 24)	SCIM III	37.5 ± 27.2	49.2 ± 31.3	−1.519	0.142	−11.7 [−26.7, 3.4]
	Proximal MAS	2.5 ± 3.8	2.1 ± 2.3	0.757	0.457	0.4 [−0.6, 1.3]
	Intermedium MAS	2.4 ± 3.3	1.7 ± 1.9	1.895	0.071	0.8 [0.0, 1.5]
	Distal MAS	2.9 ± 3.1	2 ± 1.9	2.184	0.039	0.9 [0.1, 1.7]
	Aggregated MAS	7.8 ± 9.6	5.8 ± 5.2	1.773	0.089	2 [−0.2, 4.2]
Dorsal (N = 36)	SCIM III	45.1 ± 20.5	61.3 ± 19.1	−3.397	0.002	−16.3 [−25.6, −6.9]
	Proximal MAS	5.4 ± 5	3.8 ± 3.5	3.390	0.002	1.6 [0.7, 2.5]
	Intermedium MAS	5.3 ± 4.6	3.9 ± 3.4	2.876	0.007	1.3 [0.4, 2.2]
	Distal MAS	6.6 ± 5.1	4.3 ± 3.8	4.915	0.000	2.3 [1.4, 3.3]
	Aggregated MAS	17.3 ± 13.7	12 ± 9.4	4.327	0.000	5.3 [2.9, 7.6]
Lumbar (N = 14)	SCIM III	35.5 ± 20.4	60.1 ± 22	−4.611	**<0.001**	−24.6 [−35.1, −14.2]
	Proximal MAS	3.7 ± 4.8	2.9 ± 3.6	1.669	0.119	0.8 [−0.1, 1.7]
	Intermedium MAS	3.6 ± 4	3 ± 3.7	1.749	0.104	0.6 [−0.1, 1.2]
	Distal MAS	4.7 ± 4.7	3.5 ± 3.9	1.981	0.069	1.2 [0, 2.4]
	Aggregated MAS	12 ± 12.7	9.4 ± 10.3	2.432	0.030	2.5 [0.4, 4.6]

*CI* confidence interval, *SCIM III* spinal cord independence measure (Version III), *MAS* modified Ashworth Scale.

Value is presented as mean ± standard deviation. Significant p-values are reported in bold. * Related to the paired t-test.

The subgroup analysis for etiology suggested that traumatic participants reported more improvement in terms of function and spasticity compared to non-traumatic participants (Table 6). No differences were found between traumatic and non-traumatic subgroups considering the time since the acute event (i.e., early phase, late phase; χ^2^ = 0.235, p = 0.628), ASIA at baseline (i.e., A, B, C, D; χ^2^ = 2.529, p = 0.470), and lesional level (i.e., cervical, dorsal, lumbar; χ^2^ = 1.005, p = 0.605).

**Table 6:** Secondary analysis considering the etiology (i.e., traumatic and non-traumatic), investigating differences between the baseline assessment and the discharge.

Etiology	Outcome measures	T0	T1	t-score*	p-value*	Mean change [95% CI]
Traumatic (N = 50)	SCIM III	43.1 ± 24.3	59 ± 25.4	−3.872	**<0.001**	−15.9 [−24.0, −7.9]
	Proximal MAS	4.2 ± 4.7	3.2 ± 3.3	2.887	0.006	1.0 [0.3, 1.7]
	Intermedium MAS	3.9 ± 3.9	2.8 ± 3.2	3.205	0.002	1.0 [0.4, 1.7]
	Distal MAS	5.2 ± 4.6	3.3 ± 3.2	5.203	**<0.001**	1.9 [1.2, 2.6]
	Aggregated MAS	13.4 ± 12.2	9.4 ± 8.2	4.484	**<0.001**	4 [2.2, 5.7]
Non traumatic (N = 24)	SCIM III	36.1 ± 19.3	53.3 ± 22.7	−2.481	0.021	−17.2 [−30.8, −3.6]
	Proximal MAS	4.0 ± 4.9	2.9 ± 3.2	1.976	0.060	1.1 [0.0, 2.2]
	Intermedium MAS	4.3 ± 4.9	3.4 ± 3.4	1.920	0.067	0.9 [0.0, 1.8]
	Distal MAS	4.6 ± 5.1	3.5 ± 4	2.234	0.036	1.1 [0.1, 2.0]
	Aggregated MAS	12.8 ± 14.6	9.8 ± 10.1	2.296	0.031	3.0 [0.4, 5.6]

*CI* confidence interval, *SCIM* spinal cord independence measure, *MAS* modified Ashworth Scale.

Value is presented as mean ± standard deviation. Significant p-values are reported in bold. * Related to the paired t-test.

## Discussion

This multicenter retrospective observational study evaluated the effects of gait training with the Ekso Bionics exoskeleton on spasticity and functional independence in seventy-four individuals with SCI. The results suggested a marked reduction in spasticity post-intervention in all lower limb segments. Specifically, 21.62% of the sample having no spasticity at baseline didn’t achieve any spasticity change after treatment, which can be considered as a positive outcome. While, of the rest of the sample 70,7% of the rest of the sample has gained a spasticity reduction by ≥ 1 point.

Findings corroborate previous evidence, indicating that powered exoskeletons can substantially improve mobility and functional independence, as reflected in significant gains in SCIM scores [26]. Additionally, reductions in MAS scores align with studies showing that exoskeleton use can mitigate spasticity, a frequent and disabling condition in SCI [32]. Reduced spasticity not only enhances patient comfort but also decreases risks of secondary complications such as contractures and pain [5], and the worsening of the subjects’ quality of Life [33].

Our findings align with previous studies demonstrating that exoskeleton-assisted gait training can lead to selective spasticity reduction in specific muscle groups [22, 23, 34]. A study by Juszczak et al. [23] also reported segmental differences in MAS reduction following robotic rehabilitation, highlighting the importance of individualized training protocols. Moreover, Baunsgaard et al. [9] suggested that spasticity reduction may be influenced by both lesion level and training intensity, a pattern that is evident in our findings as well. Unlike studies such as Kressler et al. [35], where limited changes in spasticity were observed, our results suggest that training dose and lesion-specific adaptations may play a crucial role in optimizing spasticity management. Further research should explore how lesion-specific variations affect long-term rehabilitation outcomes (Fig. 1).

Wearable exoskeleton-assisted gait training effects could be evaluated both as immediate effect (post-session) and after training period. Immediate positive effect was founded in previous results only considering a post-session assessment: Stampacchia et al. observed reduced spasticity measured by MAS, PENN spasm scale and NRS in in twenty-one individuals with SCI after one session of a wearable robotic training [34]. However, this study did not consider a set of training sessions. Baunsgaard et al. has considered a set of gait training sessions with multiple assessment timings on a sample of fifty-two persons with SCI [9]. They observed immediate post-session spasticity reduction, yet statistically significant pre-post training effect on spasticity was not found. In contrast, our research found positive outcomes in terms of spasticity reduction after a set training period. We also found that this positive effect on spasticity reduction is dose-dependent; therefore, the greater the number of sessions, the greater the reduction in spasticity (Table 3, Fig. 1). In addition to the statistical significance of the results, the magnitude of change observed—expressed as the mean change with 95% confidence intervals—supports the clinical relevance of the intervention. Notably, the average reduction in aggregated MAS score was 3.7 points [95% CI: 2.3, 5.1], a substantial decrease indicating a clinically meaningful impact on spasticity. Segmental improvements were also evident across proximal, intermediate, and distal muscle groups, with the greatest mean change observed distally (1.6 points), consistent with enhanced neuromotor responsiveness in lower segments. Similarly, the SCIM showed a mean improvement of 16.4 points [95% CI: 9.5, 23.4], highlighting significant gains in functional independence.

The dose-response analysis further underscores the importance of training frequency: participants receiving more than 15 sessions showed markedly higher mean changes across all outcome measures, particularly in SCIM (mean change = 25.7 [95% CI: 15.8, 35.6]) and distal MAS scores (mean change = 2.0 [95% CI: 1.1, 2.9]). Importantly, no significant differences were found between subgroups in baseline clinical variables, including etiology, ASIA grade, time since the acute event, and lesion level (all p > 0.05). Therefore, the observed dose-dependent improvements in spasticity and functional independence are unlikely to be confounded by these baseline characteristics. These findings strengthen the evidence that not only is overground exoskeleton training effective in mitigating spasticity, but that its benefits scale meaningfully with increased therapeutic exposure. Such quantitative effect sizes add valuable context for clinical interpretation and protocol planning in rehabilitation settings. The reduction in MAS scores observed in this study appeared to show a tendency toward greater improvements in individuals who completed a higher number of exoskeleton-assisted gait sessions. While this trend cannot be interpreted as evidence of a definitive dose–response effect, it is consistent with broader rehabilitation principles indicating that higher training volumes may enhance locomotor and neuroplastic outcomes [5, 13, 15]. Joint-specific patterns were also observed, with the ankle demonstrating the largest reductions (Fig. 2), potentially due to differences in segmental circuitry engagement [16] and greater biomechanical loading experienced by distal joints during exoskeleton-assisted gait [22, 36]. These findings are consistent with previous work showing that exoskeleton training can modulate spasticity differently across joints, with the ankle often displaying greater responsiveness than more proximal segments [35].

Differences in baseline clinical variables, including etiology, ASIA grade at baseline, time since the acute event, and lesion level, were assessed using chi-squared tests, and no significant differences were found between subgroups (all p > 0.05). These results suggest that the observed dose-dependent improvements in spasticity and functional independence are unlikely to be explained by baseline severity. Nevertheless, we acknowledge that our retrospective design limits full control over potential confounders, and future RCTs should consider stratification by baseline severity to confirm these dose-response effects. Understanding these patterns may help refine individualized prescriptions by identifying which joints respond more readily to robot-assisted gait training and optimizing treatment dose accordingly.

Given the retrospective pre–post design and the multidisciplinary setting of this study, it is reasonable to consider that the observed improvements reflect the combined effects of exoskeleton-assisted gait training and the broader rehabilitation program. All participants were clinically stable and maintained unchanged pharmacological therapy throughout the observation period, supporting the interpretation that the EksoGT™ training played a predominant role within this integrated therapeutic context.

Similarly, Juszczak et al. reported that in a cohort of forty-five persons with SCI, approximately one-third of participants showed a reduction in spasticity level measured by MAS at the conclusion of the trial [23]. Nevertheless, this study presents a single value of MAS without specific details regarding the lower limb segmental scores compared to our study that have considered a detailed muscle tone assessment differentiating the flexor and extensor muscles of proximal, intermedium and distal segments of more affected and less affected lower limbs [23]. However, there are some differences between the device used in this study (Indego Powered Exoskeleton) and the exoskeleton used in our study, particularly regarding the level of spinal support they provide. EksoGT™ offers greater thoracolumbar spinal support compared to Indego, enabling individuals with higher-level spinal cord injuries (SCI) to benefit from exoskeleton-assisted gait rehabilitation. This feature likely explains the specific inclusion criteria observed in the study by Juszczak et al., which recruited participants with SCI levels ranging from T3 to L2 [23]. Such considerations underscore the importance of tailoring device selection to the needs and capabilities of the target population to ensure optimal engagement and outcomes in rehabilitation protocols.

In line with our results, a study including six participants declared to perceive less spasticity after the wearable robotic training using a patient reported Likert based questionnaire [19].

Conversely, Kressler et al. reported no change in spasticity over the course of 18 training sessions with wearable robots in 3 subjects affected by SCI. In this case the negative finding should be justified by the very low rate of spasticity before training [35].

Khan et al. found mixed results regarding spasticity outcome in 12 subjects with SCI, suggesting potential differences between those with high versus low spasticity prior to training [36].

Additionally, one notable observation in this study is the potential relationship between spasticity reduction and improved functional outcomes as measured by the SCIM. The reduction of spasticity, facilitated by exoskeleton-assisted training, may lead to enhanced functional independence by improving motor control and facilitating daily activities. However, this hypothesis warrants further investigation, as current literature provides limited evidence to delineate the direct pathways linking spasticity management to SCIM improvement. These findings highlight the necessity of incorporating both clinical outcomes and patient-centered measures when evaluating the efficacy of advanced rehabilitation technologies. Although only the total SCIM III score was available for this retrospective analysis, the Mobility subscale is clinically the most likely to reflect changes associated with spasticity reduction and gait-related interventions. Future prospective studies should include subscale-level analyses to determine whether improvements are primarily driven by the Mobility domain, which would strengthen clinical interpretation and protocol design.

This study offers a novel contribution by specifically focusing on the spasticity outcomes associated with exoskeleton-assisted gait training, which remains an under-explored area in SCI rehabilitation research. In fact, a recent review found that only 14 of 48 studies regarding powered exoskeleton has explored the domain of spasticity [26].

While prior studies primarily focus on ambulation and functional recovery [3, 20], our findings provide new insights into how exoskeleton use directly affects spasticity reduction. By emphasizing this less-studied outcome, we introduce a broader understanding of the therapeutic benefits of exoskeletons in SCI management, potentially impacting quality of life and reducing reliance on adjunct therapies for spasticity management [23]. In addition, unlike traditional gait training or stationary robotic systems, overground exoskeletons simulate near-physiological walking patterns, promoting natural sensory feedback and dynamic movement. These features facilitate neuromuscular re-education by encouraging proprioceptive input, which is critical for balance recovery and coordinated motor control [35]. Moreover, interaction with the surrounding environment during gait training offers additional external stimuli, which reinforces the therapeutic benefits, as suggested in studies on the importance of sensory-motor feedback in SCI rehabilitation [13].

A key strength of this study is its multicenter and retrospective observational design, which increases the generalizability of findings across various rehabilitation sites. This design enables analysis of real-world data collected during routine clinical care, reflecting the practical application of exoskeleton-assisted rehabilitation. Additionally, the study’s observational design leverages real current use of the device-, contributing clinical evidence on treatment dose as a modifier of rehabilitation outcomes, an area with limited empirical guidance. This study provides valuable insights into integrating exoskeletons into clinical practice and highlights the need for evidence-based guidelines to optimize their use in SCI rehabilitation [23] addressed to improve standards of care for people with SCI [32]. Our results underscore the importance of structured training regimens that emphasize high frequency and intensity, which are vital for maximizing the benefits of robotic therapy.

Although individuals with L2–L3 lesions were included in the study, it is acknowledged that lesions at these levels often involve lower motor neuron pathways and may not present with spasticity. In fact, most participants in this subgroup exhibited no spasticity at baseline, and consequently, no measurable change was observed post-intervention. Their inclusion, however, reflects the clinical reality and diversity of individuals undergoing exoskeleton-assisted gait training. Therefore, the results concerning this subgroup should be interpreted in the context of the specific neuroanatomical characteristics of their lesions.

Moving forward, further research—particularly randomized controlled trials—will be essential to validate these findings and explore the long-term effects of exoskeleton-assisted rehabilitation and ensure comprehensive data collection on additional outcomes, such as pain and quality of life (QoL). Moreover, further investigation into the multiple domain effects (e.g., costs, acceptance, and other issues important for integration into the health domain) [37] of exoskeleton use on SCI rehabilitation is recommended.

It should be acknowledged that, as a retrospective pre–post study, the observed effects likely result from the combined influence of exoskeleton-assisted training and concurrent multidisciplinary rehabilitation. However, all participants were clinically stable and maintained consistent pharmacological regimens, supporting the interpretation that the improvements are plausibly related to the integration of robotic gait training within standard care. The responsiveness of spasticity to gait training may be influenced by differences in corticospinal tract integrity and the presence of residual voluntary motor activity, particularly relevant when comparing AIS A and AIS B individuals. The absence of motor ZPP data in our dataset prevents subgroup stratification based on residual motor pathways. Future studies should incorporate neuroanatomical markers of motor preservation to better characterize differential treatment responsiveness.

This study highlights the significant impact of overground wearable powered exoskeletons on improving functional independence and reducing spasticity in individuals with spinal cord injury (SCI). Greater reductions in spasticity and improvements in functional independence were observed in participants who completed a higher number of exoskeleton-assisted gait training sessions (>15), demonstrating a dose-dependent effect. This approach addresses critical aspects of rehabilitation, including the management of spasticity and the enhancement of overall quality of life.

## Data Availability

The datasets generated and analyzed during the current study are available from the corresponding author upon reasonable request.
